# Physicochemical Properties and Sensory Quality of Marine Plant–Based Seasoning From Red Seaweed (*Gracilaria* sp.) Combined With Mushroom (*Agaricus bisporus*)

**DOI:** 10.1155/ijfo/2588096

**Published:** 2026-10-08

**Authors:** Muhammad Yusuf, Sidney Akumu Odongo, Siti Zaharah Rosli

**Affiliations:** ^1^ Department of Food Technology Faculty of Agricultural Science and Technology Muhammadiyah University of Semarang Central Java Semarang Indonesia; ^2^ Department of Diagnostic and Allied Health Sciences Faculty of Health and Life Science Management and Science University Shah Alam Malaysia msu.edu.my

**Keywords:** *Agaricus bisporus*, *Gracilaria* sp., plant-based, seasoning, umami

## Abstract

This study examined the creation of a plant‐based seasoning powder derived from red seaweed (*Gracilaria* sp.) enhanced with white button mushroom (*Agaricus bisporus*) powder. Four formulations were tested: a control (0% mushroom) and three with 10%, 20%, and 30% mushroom content. Physicochemical analysis revealed that increasing mushroom levels significantly reduced water content, from 3.976% in the control to 2.852% at 30% mushroom (*p* < 0.001), whereas all products remained within a safe microbial stability range (< 4%). Glutamic acid levels rose proportionally with mushroom addition, indicating a boost in savory compounds. Sensory evaluations employed the Friedman test, followed by Wilcoxon′s signed‐rank post hoc analysis. Results showed that texture was consistent across treatments (*p* = 0.6444), but taste, aroma, and color scores significantly improved with mushroom inclusion. The formulation with 30% mushroom (P3) received the highest hedonic scores for taste (4.10) and a notably superior aroma (4.00) compared to the seaweed‐only control. These findings suggest that fortifying with 30% mushroom harnesses umami synergy to transform a marine‐based seaweed substrate into a highly acceptable, shelf‐stable, clean‐label savory seasoning.

## 1. Introduction

The sensation of flavor reflects the complex integration of aroma, taste, and texture from food or a food component; it is a major determinant of food palatability, the extent to which a food is accepted or rejected [1]. Flavor affects dietary choices, preferences, palatability, and daily consumption decisions in humans both before and after food ingestion [2]. In the food industry, flavor enhancers and additives play a pivotal role, providing numerous benefits, including improving nutritional quality, enhancing sensory properties, extending shelf life, and improving overall quality [3].

In Asia, monosodium glutamate (MSG) is the most common flavor enhancer used in home cooking, particularly in countries like China, Japan, and Thailand [4]. Its use has sparked controversy, as repeated exposure to MSG has been linked to obesity, cardiometabolic issues, liver, kidney, neurological, and reproductive toxicity. However, comprehensive human studies at typical intake levels are limited, and more well‐designed trials and cohort studies are necessary [5–7].

Consumer demand for “clean‐label” ingredients has increased over the years [8]. Consumers are now urging manufacturers to substitute MSG with plant‐based “clean‐label” seasonings that provide a robust flavor. *Agaricus bisporus*, or button mushrooms, are commonly used as natural flavoring agents, especially for their umami and kokumi (mouth‐filling) qualities in savory dishes. Mushrooms are low in calories, fat, sodium, and cholesterol but rich in protein, carbohydrates, and dietary fibers like chitin and *β*‐glucans [9–11]. Mass spectrometry imaging of edible mushrooms revealed that flavor molecules such as amino acids, sugars, nucleotides, and organic acids are unevenly distributed in the cap, gills, and stipe, allowing for targeted extraction of flavor concentrates [12]. This makes mushrooms an ideal choice for developing natural seasonings.

The use of macroalgae or fungi individually as flavoring agents has been studied, but integrating red seaweed with button mushroom offers a unique biotechnological benefit. Edible red seaweed is abundant in free umami amino acids like L‐glutamate and L‐aspartate, which provide a savory oceanic flavor [13]. *A. bisporus* contains higher levels of purine 5 ^′^‐ribonucleotides, such as GMP and AMP, unevenly distributed in its gills [12]. Combining marine and terrestrial substrates enhances flavor beyond simple addition; instead, they interact synergistically to activate human T1R1/T1R3 umami receptors [14, 15]. This molecular synergy greatly reduces the human detection threshold for savoriness, offering a scientifically supported way to replace MSG without compromising food palatability [1, 4].

Red seaweed (*Gracilaria* sp.) is an edible seaweed traditionally used in salads, soups, and desserts throughout Asia and the Caribbean. It has recently gained attention as a flavor enhancer, seasoning, and functional ingredient in modern foods [16]. *Gracilaria* species are rich in umami amino acids like aspartic and glutamic acids, which give the algae its distinctive flavor and serve as natural taste enhancers [13]. Furthermore, *Gracilaria* powders contain high levels of dietary fiber, minerals, and bioactive polysaccharides with antioxidant and prebiotic benefits, supporting their function as a flavoring agent rather than just a carrier [17].

Optimizing this hybrid matrix will directly advance the paradigms of clean label and marine circular bioeconomy. Recently, consumers are increasingly demanding transparent, minimally processed ingredients free from synthetic flavor enhancers [3, 8]. The utilization of *Gracilaria* sp. meets this demand by serving not only as an inert material carrier but also as a functional macroalgal base that provides dietary fibers, trace minerals, and prebiotic polysaccharides [16, 17]. From a biotechnological and environmental perspective, cultivating red seaweed supports coastal bioeconomies through sustainable aquaculture practices, as mushrooms offer low‐calorie, nutrient‐dense biomass [9, 10]. Transforming these renewable marine resources and widely cultivated terrestrial fungi into high‐value seasoning powders creates a scalable blueprint for waste reduction, low‐carbon food processing, and resilient plant‐based food systems. This research is aimed at studying physicochemical properties and sensory qualities.

## 2. Materials and Methods

The red seaweed *Gracilaria* sp. type was obtained from ponds in Tugu Village, Sayung District, Demak Regency. Button mushrooms (*A. bisporus*) were bought from Indomaret Supermarket in Semarang, along with other additives such as sugar, pepper powder, orange, and garlic powder. Other materials used for the chemical analysis included glutamic acid standards, ninhydrin, ethanol, and distilled water.

### 2.1. Preparation of Red Seaweed Powder

Red seaweed was thoroughly washed with clean water to remove surface impurities. It was then soaked in 10 mL of water per gram of seaweed, and 5% orange juice by weight of the seaweed was added to neutralize alkaline compounds, reduce the pungent “fishy” smell, and help break down its complex polysaccharides. The seaweed was soaked for 30 min, then cut into smaller pieces, and boiled for 5 min. It was dried in a cabinet dryer at 60°C until brittle. It was blended into a powder and then sieved through a 60‐mesh sieve. The powder was stored in a vacuum‐sealed plastic bag for further use.

### 2.2. Preparation of Button Mushroom (*A. bisporus*) Powder

*A. bisporus* preparation followed [18] with a few modifications. Fresh white button mushrooms, free of defects, were washed in running water and then cut into 2‐mm slices with a manual slicer. The sliced mushrooms were placed in a cabinet dryer at 60°C and dried for 4–5 h until brittle. Once dried, they were ground into a powder and sieved through a 60‐mesh sieve. The powder was stored in a vacuum‐sealed bag for future use.

### 2.3. Flavoring Powder Preparation

Four formulations were developed by blending red seaweed *Gracilaria* sp. with *A. bisporus* at different concentrations (0%, 10%, 20%, and 30%). Each batch included 50 g of red seaweed (*Gracilaria* sp.) mixed with *A. bisporus* according to treatment levels. The recipes also contained salt, sugar, pepper powder, and garlic, as listed in Table 1.

**Table 1:** Formulation of red seaweed and mushroom seasoning powder.

Material	Treatment			
	P1	P2	P3	P4
Red seaweed (g)	50	50	50	50
*Agaricus bisporus* (%)	0	10	20	30
Sugar (%)	5	5	5	5
Ground pepper (%)	5	5	5	5
Garlic powder (%)	5	5	5	5

### 2.4. Mineral Analysis

Mineral analysis was performed according to [19] with modifications. One gram of flavoring powder was subjected to digestion with nitric acid (HNO_3_) and hydrochloric acid (HCl) in an 8:2 ratio at 180°C for 40 min. The mineral was dissolved in a standard flask in 25 mL of deionized water. The concentrations of the minerals Ca, Na, P, Mg, K, Fe, Cu, Zn, and Mn were quantified using atomic absorption spectrophotometry.

### 2.5. Glutamic Acid

To assess the umami potential of *Gracilaria* sp. and mushroom seasoning powder, glutamic acid levels were determined using a spectrophotometric method based on the ninhydrin reaction. The analysis began by establishing a standard curve according to the protocol of [20] using standard glutamate solutions at concentrations of 50, 100, 200, 300, 400, and 500 ppm. For each standard, a 2‐mL aliquot is reacted with 1 mL of ninhydrin solution and heated until a characteristic color change occurs. After heating, the solutions were cooled for 15 min, stabilized with 2 mL of ethanol to ensure homogeneity, and allowed to stand for approximately 10 min before absorbance was measured at 570 nm. The resulting linear regression (Equation 1) is as follows:

(1) y=ax+b,

where *Y* is the sample absorbance, *X* is the concentration (parts per million), *A* is the intercept, and *B* is the slope of the line.

The testing process, adapted from [21] with modifications, involved dissolving 0.5 g of flavoring powder in 50 mL of distilled water. A 2‐mL sample of this mixture was transferred to a test tube, mixed with 2 mL of ninhydrin solution, and heated in boiling water for 15 min to initiate the colorimetric reaction. After 15 min of cooling, the absorbance was measured at 570 nm using a UV‐Vis spectrophotometer. The glutamic acid concentration was then determined by entering the absorbance into the standard curve equation, accounting for the dilution factor (*F* *p*) and the sample weight. This approach provided an accurate measure of how mushroom fortification enhances the natural amino acid content of the seaweed‐based seasoning.

### 2.6. Water Content

The moisture content of the seasoning powder was evaluated according to [22] using the thermogravimetric (oven‐dry) method. Clean porcelain crucibles were rinsed with distilled water and dried in an oven at 105°C for 15 min. After cooling in a desiccator for 15 min to reach room temperature, the crucibles were weighed to determine their initial weight (*W*). A 1‐g sample of seasoning powder was added to each crucible and weighed (*W*1). The crucibles with samples were then heated at 105°C in a drying oven for 2 h. After heating, the samples were cooled in a desiccator for 15 min and reweighed (*W*2). This drying and weighing process was repeated until a constant weight was achieved, indicating that all moisture had been removed. The water content percentage was then calculated using this formula:

(2) water content=W12−WW1−W×100,

where *W* is the mass of the empty crucible, *W*1 is the mass of the empty crucible + wet sample, and *W*2 is the mass of the empty crucible + dried sample.

### 2.7. Amino Acids

Amino acid analysis was performed following [23] with some modifications. Proteins were first extracted from the samples using the Kjeldahl method before hydrolysis. The analysis utilized a high‐performance liquid chromatography (HPLC) system (Shimadzu RF‐20A, Kyoto, Japan), adhering to the [22] protocol. The hydrolysis process involved treating 3 mg of the flavoring sample with 5 mL of 0.01 N HCl, then incubating it in an oven at 110°C for 24 h. After hydrolysis, the mixture was filtered through Millipore paper, and 10 *μ*L of the sample was derivatized with 25 *μ*L of OPA reagent. A 5‐*μ*L aliquot of the derivatized sample was then injected into the HPLC for amino acid separation. The HPLC conditions included a reversed‐phase C18 column, a fluorescence detector, a column temperature of 28°C, and a mobile phase flow rate of 1 mL/min. The mobile phase comprised Buffer A (0.025 M Na‐acetate, 0.05% Na‐EDTA, 9% methanol, and 1% THF) and Buffer B (95% methanol). The amino acid concentrations were determined based on standard curves generated from peak area measurements.

### 2.8. Sensory Evaluation

The sensory properties of the seaweed and mushroom seasoning powders were assessed through a hedonic test to gauge consumer acceptance of color, aroma, taste, and texture. The evaluation involved formulations with different mushroom powder levels (0%, 10%, 20%, and 30%). For testing, the powders were dissolved in broth at a 1:50 (*w*/*v*) ratio.

The test involved 50 semitrained panelists (students) from the food technology department at Universitas Muhammadiyah Semarang. They were presented with four liquid samples, each labeled with a unique three‐digit randomized code. The evaluation was conducted in individual sensory booths with consistent lighting conditions. Panelists rated their preferences using a 5‐point hedonic scale, from 1 (*dislike very much*) to 5 (*like very much*). The collected data were analyzed statistically. The Friedman test was used initially to detect significant differences among the treatment levels. If the Friedman test showed a significant result, a post hoc Wilcoxon signed‐rank test was conducted to identify specific differences between pairs of samples.

### 2.9. Statistical Analysis

A completely randomized design (CRD) with one factor, involving four treatments and six repetitions, was used. All data, both physicochemical and sensory, were analyzed via one‐way ANOVA. When significant differences were detected (*p* < 0.05), Duncan′s multiple range test (DMRT) was used to rank the treatment means. Data were analyzed with MATLAB R2025b. Results were shown as mean ± standard deviation. Statistical significance was set at *p* < 0.05.

## 3. Results and Discussion

### 3.1. Glutamic Acid Analysis

The analysis of glutamic acid levels across the four treatments (0%, 10%, 20%, and 30%) of mushroom fortification demonstrated a significant linear increase in umami‐contributing compounds. The ANOVA of the data indicated a significant effect (*p* < 0.05) of mushroom fortification on glutamic acid levels in the seasoning powder. The substantial increase from the control (0.949%) to the highest treatment level (2.893%) confirmed that mushrooms are a potent natural source of glutamic acid, significantly enhancing the savory profile of the red seaweed base. Many edible mushrooms contain high levels of glutamic acid, aspartic acid, and 5 ^′^‐ribonucleotides (GMP, IMP, and AMP), which are all important umami compounds [24, 25]. Similarly, seaweeds and microalgae add glutamate, aspartate, and 5 ^′^‐nucleotides, contributing to an “oceanic” savory flavor [26, 27]. L‐Glutamate and 5 ^′^‐nucleotides act synergistically at the T1R1/T1R3 umami receptor, greatly enhancing taste when combined compared with glutamate alone [14, 15]. Increased mushroom content or enzymatic hydrolysis in various systems significantly raises free glutamate and EUC levels, as shown by the progression from P0 to P3 in Figure 1 [28, 29]. In the study by [30], enzymatic hydrolysates of mushroom mycelium increased free glutamate levels by more than 20‐fold, clearly boosting umami flavor in broth.

**Figure 1:**
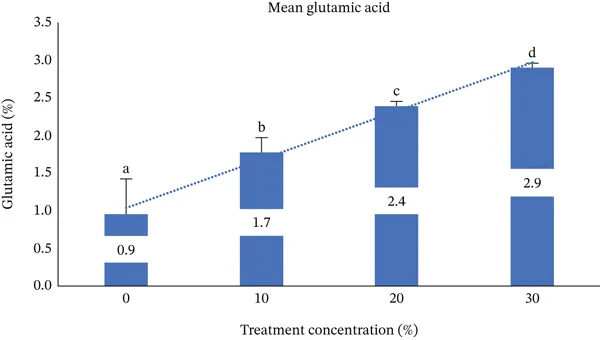
Mean glutamic acid concentration.

According to [31], purine nucleotides (IMP, GMP, and AMP) significantly lower glutamate detection thresholds and boost umami intensity in humans. This aligns with higher DMRT scores at 20%–30% mushroom, where both glutamate and nucleotides are elevated. Mushroom‐based flavorings are increasingly being proposed as natural alternatives to MSG and animal‐based flavorings in meat products and savory seasonings [25, 28]. Umami ingredients (Glu, nucleotides, and umami peptides) can enhance saltiness and overall flavor, enabling a 20%–30% reduction in sodium [24].

### 3.2. Mineral Content

The progressive integration of button mushroom (*A. bisporus*) powder significantly altered the elemental composition of the macroalgal seasoning matrix, establishing a clear accretion path for crucial macrominerals.

The clearest observed response was in potassium and phosphorus, which increased stepwise from P0 to P3. Potassium rose from 6.79 ± 0.01 to 14.11 ± 0.01, whereas phosphorus increased from 0.29 ± 0.01 to 2.82 ± 0.01 (Table 2), suggesting that mushroom inclusion disproportionately contributed minerals already reported as prominent in button mushroom, particularly potassium and phosphorus [32, 33]. This pattern was consistent with broader compositional surveys showing that potassium is typically the dominant mineral in cultivated *A. bisporus*, although the absolute concentrations varied markedly by origin, substrate, and market source [33–35]. Similarly, the same situation is observed across *Gracilaria* species, where potassium is frequently among the main macroelements, but it is not consistent across all taxa or collection sites; some datasets instead have highlighted calcium or sodium as the primary fraction [36–38].

**Table 2:** Mineral mass distribution (milligrams per 1‐g sample) of flavoring powder.

Mineral	P0 (0%)	P1 (10%)	P2 (20%)	P3 (30%)	*p*value
Potassium (K)	6.79 ± 0.01^a^	9.31 ± 0.01^b^	11.69 ± 0.01^c^	14.11 ± 0.01^d^	< 0.0001
Calcium (Ca)	2.60 ± 0.01^a^	2.67 ± 0.01^b^	2.71 ± 0.01^c^	2.78 ± 0.01^d^	< 0.001
Phosphorus (P)	0.29 ± 0.01^a^	1.12 ± 0.01^b^	1.99 ± 0.01^c^	2.82 ± 0.01^d^	< 0.0001
Sodium (Na)	1.44 ± 0.01^a^	1.53 ± 0.01^b^	1.58 ± 0.01^c^	1.67 ± 0.01^d^	< 0.001
Magnesium (Mg)	0.36 ± 0.01^a^	0.36 ± 0.01^a^	0.40 ± 0.01^b^	0.42 ± 0.01^c^	< 0.01
Iron (Fe)	0.28 ± 0.01^a^	0.27 ± 0.01^a^	0.30 ± 0.01^b^	0.29 ± 0.01^b^	< 0.05
Zinc (Zn)	0.03 ± 0.01^a^	0.04 ± 0.01^a^	0.09 ± 0.01^b^	0.10 ± 0.01^b^	< 0.001
Copper (Cu)	0.01 ± 0.01^a^	0.03 ± 0.01^b^	0.02 ± 0.01^ab^	0.05 ± 0.01^c^	< 0.05
Manganese (Mn)	0.02 ± 0.01^a^	0.01 ± 0.01^a^	0.04 ± 0.01^b^	0.03 ± 0.01^b^	< 0.05

*Note:* Values are reported as mean ± standard error (*n* = 6). Means within the same row with different superscript letters (a, b, c, and d) differ significantly according to Duncan′s multiple range test (DMRT) at *p* < 0.05. P0 = 0% mushroom, P1 = 10% mushroom, P2 = 20% mushroom, and P3 = 30% mushroom.

Calcium, sodium, and magnesium levels increased notably but modestly. Calcium increased from 2.60 ± 0.01 to 2.78 ± 0.01, sodium from 1.44 ± 0.01 to 1.67 ± 0.01, and magnesium from 0.36 ± 0.01 to 0.42 ± 0.01, with no significant difference in magnesium between P0 and P1. This limited response is understandable since some studies suggest that *A. bisporus* can have relatively high calcium, magnesium, and sodium levels, and *Gracilaria* already maintains substantial baseline mineral levels, which may restrict additional enrichment in blended systems [36, 38, 39].

The distribution of trace elements was more variable than that of macroelements. Zinc exhibited a clear increasing trend, rising from 0.03 ± 0.01 in P0 to 0.10 ± 0.01 in P3, with significant differences observed between P2 and P3 compared to P0 and P1. This supports the idea that mushrooms can provide zinc for fortified foods and that fungal matrices are effective carriers for trace minerals [40–42]. Copper levels also increased overall but followed a nonlinear pattern, with P2 showing an intermediate level between P1 and P3. Manganese and iron experienced minor yet significant fluctuations without a consistent upward trend; iron went from 0.28 ± 0.01 to 0.30 ± 0.01 before slightly decreasing to 0.29 ± 0.01, whereas manganese decreased at P1, increased at P2, and then leveled off at P3. These results indicate that the incorporation of trace minerals into the seasoning matrix depends not only on their content in mushroom powder but also on complex interactions within the matrix, background algal composition, and species‐specific accumulation as seen in both mushrooms and seaweeds [36, 37, 39].

### 3.3. Water Content Analysis

The statistical analysis confirmed that adding mushroom powder significantly influenced the water content of the red seaweed–mushroom seasoning powder. As the concentration of mushroom powder increased, the water content decreased notably. In Figure 2, P0 showed the highest water content at 3.976%, whereas increasing mushroom content to P3 (30%) reduced water content to its lowest level of 2.852%. ANOVA results demonstrated a highly significant difference (*p* < 0.001) among the treatment groups. These results are consistent with the standard dried seasoning threshold (< 10%), which is ideal for microbial stability and an extended shelf life.

**Figure 2:**
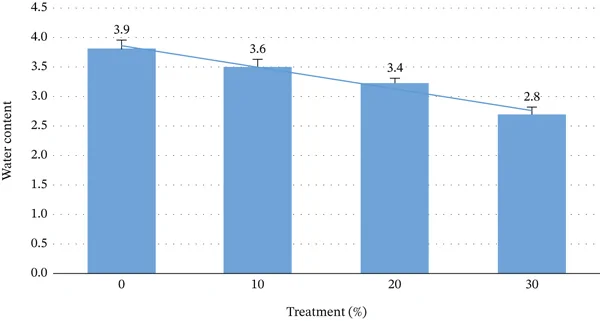
Water content per treatment.

The water content of the developed seasoning formulation ranged from 3.976% to 2.852%, falling within the safety margins required for a stable dehydrated powder. These levels are critical, as they inhibit microbial growth, enzymatic activity, and degradative reactions such as lipid oxidation and nonenzymatic browning [43, 44]. Safe storage of low‐moisture foods is achieved when moisture is below 10%, effectively preventing mold and mycotoxin formation [45, 46]. Although microbial and formal shelf‐life testing was not conducted in this study, the extremely low moisture levels suggest high physical and chemical stability. Maintaining this dryness is essential to prevent caking and to preserve the free‐flowing texture necessary for seasoning products [47, 48].

The observation (Figure 2) that the seaweed‐only control (P0) had the highest moisture, which then significantly decreased with each 10% increase in mushroom powder, aligns with the different biochemical properties of the raw materials. Red seaweeds, such as *Gracilaria* spp., are rich in hydrocolloids, including agar, which have strong water‐binding capacity and hygroscopic properties [49, 50]. Dried mushroom powders primarily consist of insoluble fibers and proteins, which facilitate more efficient water removal during drying [51, 52]. This explains why substituting seaweed with powder in P1–P3 led to a gradual decrease in total water content. A 30% mushroom treatment with the lowest moisture is expected to offer the best resistance to clumping and maximum stability, especially if stored in moisture‐free packaging to prevent water reabsorption from humid environments [46, 53].

### 3.4. Amino Acid

The quantification of total amino acids via HPLC revealed a highly significant (*p* < 0.05) restructuring of the seasoning′s nutritional and flavor‐active backbone (Table 3). Glutamic acid and aspartic acid showed the most pronounced formulation response. They increased from 11.45 to 36.54 mg/g and from 9.82 to 14.65 mg/g, respectively, across all treatments.

**Table 3:** Total amino acid profile via hydrolyzed HPLC (milligrams per 1.0 g of dry sample) across formulations.

Amino acid	P0 (0)	P1 (10)	P2 (20)	P3 (30)	*p*value
Glutamic acid (Glu)	11.45 ± 0.03^a^	19.82 ± 0.04^b^	28.15 ± 0.05^c^	36.54 ± 0.06^d^	< 0.0001
Aspartic acid (Asp)	9.82 ± 0.02^a^	11.44 ± 0.03^b^	13.02 ± 0.03^c^	14.65 ± 0.04^d^	0.0001
Leucine (Leu)	4.22 ± 0.01^a^	5.31 ± 0.02^b^	6.44 ± 0.02^c^	7.56 ± 0.02^d^	< 0.001
Lysine (Lys)	3.16 ± 0.01^a^	4.12 ± 0.02^b^	5.07 ± 0.02^c^	6.02 ± 0.03^d^	< 0.001
Valine (Val)	3.41 ± 0.01^a^	4.17 ± 0.01^b^	4.92 ± 0.02^c^	5.67 ± 0.02^d^	< 0.01
Isoleucine (Ile)	2.61 ± 0.01^a^	3.27 ± 0.02^b^	3.91 ± 0.02^c^	4.57 ± 0.02^d^	< 0.01
Phenylalanine (Phe)	2.91 ± 0.01^a^	3.47 ± 0.01^b^	4.02 ± 0.02^c^	4.57 ± 0.02^d^	< 0.05
Threonine (Thr)	3.11 ± 0.01^a^	3.57 ± 0.02^b^	4.07 ± 0.02^c^	4.52 ± 0.02^d^	< 0.05
Arginine (Arg)	4.82 ± 0.02^a^	5.17 ± 0.02^a^	5.52 ± 0.03^b^	5.87 ± 0.03^b^	< 0.05
Alanine (Ala)	5.21 ± 0.03^a^	5.42 ± 0.03^a^	5.62 ± 0.03^a^	5.82 ± 0.04^a^	0.2415 (ns)
Glycine (Gly)	4.46 ± 0.01^a^	4.72 ± 0.02^a^	4.97 ± 0.02^b^	5.22 ± 0.02^b^	< 0.05
Serine (Ser)	3.21 ± 0.01^a^	3.62 ± 0.02^b^	4.02 ± 0.02^c^	4.42 ± 0.02^d^	< 0.01
Tyrosine (Tyr)	1.91 ± 0.01^a^	2.17 ± 0.02^ab^	2.42 ± 0.02^b^	2.67 ± 0.02^c^	< 0.05
Proline (Pro)	2.81 ± 0.02^a^	2.97 ± 0.02^a^	3.12 ± 0.02^a^	3.27 ± 0.03^a^	0.1873 (ns)
Methionine (Met)	0.66 ± 0.01^a^	0.77 ± 0.01^b^	0.88 ± 0.01^b^	1.01 ± 0.01^c^	< 0.01

*Note:* Values are reported as the mean ±standard deviation (*n* = 6). Within each row, values with different superscript letters (a, b, c, and d) differ significantly between formulation levels, according to Duncan′s multiple range test (*p* < 0.05). Superscript combinations (e.g., ab) indicate values that are not significantly different from either of the corresponding letter groups.

Abbreviation: ns, not significant.

The cautious inclusion of mushrooms may have increased the acidic amino acids responsible for seasoning. However, the extent of this increase should be evaluated in conjunction with the analytical method employed. Acid hydrolysis transforms glutamate and asparagine into glutamate and aspartate during total amino acid analysis [54, 55]. Moreover, *A. bisporus* powder has been reported to contain glutamate as a dominant free amino acid, with aspartate serving as a major contributor to umami [56]. In macroalgae such as *Gracilaria*, Machado et al. [54] identified aspartic acid as the most abundant nonessential amino acid, followed by glutamic acid. Tropical seaweeds exhibit nonessential amino acid patterns in which Asp + Glu together account for 25.2%–29.5% of the amino acid content [57].

Another pattern identified was the synchronized increase in essential amino acids such as leucine, lysine, valine, and isoleucine. All of these showed significant growth from P0 to P3. This gradual and moderate increase suggests that adding mushrooms may help improve the balance of essential amino acids in the mixture rather than automatically enhancing overall protein quality [56, 58].

This was supported by a comparison with existing seaweed literature. Macroalgae can offer complete essential amino acid profiles, but methionine is often limiting, and some seaweeds have been reported to have deficiencies in lysine and leucine [54, 57]. In contrast, *A. bisporus* contains all nine essential amino acids in powder form, as shown in a recent study. Mushrooms, more broadly, are considered good sources of most essential amino acids compared to common vegetables [56]. The increase in methionine from 0.66 to 1.01 mg/g (Table 3) is thus significant because methionine is a frequently limiting amino acid in seaweed datasets, including *Gracilaria salicornia* [59].

### 3.5. Sensory Evaluation

The sensory evaluation results, analyzed using the Friedman test and Wilcoxon signed‐rank post hoc test, showed that replacing red seaweed with mushroom powder changed the outcome. These nonparametric tests were crucial for hedonic data because they handle the subjective, ordinal nature of human preferences, as shown in Table 4.

**Table 4:** Sensory evaluation profile mapping of red seaweed (*Gracilaria* sp.) seasoning powder formulated with varying inclusion levels of button mushroom (*Agaricus bisporus*) powder.

Sensory attribute	P0 (0% mushroom)	P1 (10% mushroom)	P2 (20% mushroom)	P3 (30% mushroom)	Friedman (*p*value)
Color	3.56 ± 0.81^a^	3.68 ± 0.71^a^	3.94 ± 0.74^b^	4.26 ± 0.69^c^	0.0001
Aroma	3.40 ± 0.83^a^	3.58 ± 0.76^ab^	3.80 ± 0.67^b^	4.20 ± 0.67^c^	< 0.0001
Taste	3.08 ± 0.73^a^	3.46 ± 0.76^b^	3.96 ± 0.64^c^	4.48 ± 0.58^d^	< 0.0001
Texture	3.78 ± 0.74^a^	3.82 ± 0.72^a^	3.86 ± 0.67^a^	3.88 ± 0.72^a^	0.6549

*Note:* Values are reported as mean ± standard deviation (*n* = 50 panelists) on a 5‐point hedonic scale. Within each row, values with different superscript letters (a, b, c, and d) differ significantly according to the Wilcoxon signed‐rank post hoc test (*p* < 0.05), following a significant Friedman test. Superscript combinations (e.g., ab) indicate scores that are not significantly different from either letter group. Treatment codes: P0 = 0% mushroom, P1 = 10% mushroom, P2 = 20% mushroom, and P3 = 30% mushroom powder.

A significant change in taste was observed (*p* = 0.0001), as the control group (P0), which consisted solely of a red seaweed base, had the lowest preference score of 3.08. However, the addition of mushroom powder led to a notable increase in scores. The Wilcoxon test indicated that P3 (30% mushroom) was statistically superior to all other treatments, with a score of 4.48. This finding helps to scientifically support the concept of umami synergy.

Aroma scores improved from 3.00 (P0) to 4.00 (P3). Seaweed often carries a distinct marine or fishy scent due to volatile sulfur compounds and bromophenols [60]. At lower fortification levels (P1 and P2), the improvement in aroma was not statistically significant compared with the control (*p* > 0.05).

The visual appeal reached its peak at P2 with 20% mushroom, scoring 4.00 (color). Raw red seaweed powder usually has a dull, brownish‐purple or pale green shade that may not immediately evoke “seasoning” in consumers [61]. Adding mushroom powder introduces warmer, golden‐brown hues, often caused by mild enzymatic browning during drying. Notably, at 30% (P3), the color score slightly decreased to 3.75, indicating an aesthetic “sweet spot” at 20% before the powder becomes too dark.

Furthermore, the Friedman test for texture yielded a nonsignificant *p* value (0.6549). This was favorable for product development, as it suggests that replacing seaweed with mushroom powder does not affect the powder′s physical mouthfeel, grittiness, or solubility.

The sensory evaluation of the red seaweed‐based seasoning revealed that mushroom fortification consistently improved specific attributes, with the most significant effects observed at a 30% inclusion level. The Friedman test was suitable for this ordinal hedonic data and confirmed that mushroom concentration notably influenced perceived color, aroma, and taste, whereas texture remained unchanged across all formulations (*p* = 0.6549). This texture stability aligns with findings in other mushroom‐enriched systems where the carrier matrix is sufficiently robust to support moderate to high mushroom levels without affecting mouthfeel [62–64]. The lack of significant differences in texture indicates that up to 30% mushroom powder can be incorporated without causing “grittiness” or undesirable structural changes, a trend often seen when increasing mushroom content in foods where flavor and appearance are more easily altered than the fundamental structure [64, 65].

Taste proved to be the most distinguishing feature and is key to the seasoning′s role as a flavor enhancer. The 30% mushroom formulation (P3) received a significantly higher hedonic score (4.48) than all other versions, indicating a clear shift in consumer preference at the highest fortification level (*p* < 0.01). This aligns with extensive scientific research indicating that mushrooms naturally contain high levels of glutamic acid, aspartic acid, and 5 ^′^‐nucleotides such as 5 ^′^‐GMP and 5 ^′^‐AMP, which together produce strong umami and savory flavors [24, 66, 67]. Past research on mushroom extracts shows that higher amounts of these umami compounds can significantly boost saltiness and overall savoriness in model soups, often allowing the products to compete with MSG‐based controls [68, 69]. The excellent performance of the P3 formulation suggests that the 30% inclusion likely placed the seasoning within an optimal “umami range,” enhancing the synergistic effect between mushroom nucleotides and the seaweed′s base glutamate [70, 71].

Color and aroma responses varied in a graded manner, requiring specific thresholds for statistical significance. Adding mushroom (10%–30%) notably enhanced color scores over the seaweed control (*p* < 0.05), indicating even low levels can shift the visual impression from the dull marine hues of plain seaweed to a more familiar golden‐brown, seasoning‐like appearance [63, 65]. For aroma, only the 30% formulation (P3) differed significantly from the control, suggesting that a threshold of mushroom volatiles such as 1‐octen‐3‐one and Maillard‐based pyrazines is necessary to dominate the scent profile [71, 72]. At lower levels (10%–20%), earthy and meaty notes may only partly mask the seaweed′s marine essence, but at 30%, these compounds are strong enough to shift perception toward a savory mushroom aroma [68, 73]. Therefore, P3 strikes an optimal balance across all sensory aspects, effectively using mushroom components to transform a marine‐flavored seaweed base into an appealing, savory seasoning [62, 64].

## 4. Conclusion

Incorporating white button mushroom (*A. bisporus*) into a red seaweed (*Gracilaria* sp.) base successfully created a plant‐based seasoning with enhanced physicochemical and sensory qualities. The study showed that mushroom addition is a key design factor, notably reducing moisture content and increasing shelf stability and caking resistance. Specifically, the formulation with 30% mushroom powder (P3) strikes an optimal balance, offering a strong umami flavor that outperforms lower levels. Statistical tests like Friedman and Wilcoxon confirmed that while the seaweed′s physical texture remains intact, its aroma and taste profiles are notably altered. The 30% level effectively masks the seaweed′s marine notes with savory, earthy mushroom aromas. Overall, the P3 formulation is a sensory‐optimized, low‐moisture, glutamic acid‐rich alternative to artificial flavor enhancers, supporting the move toward “clean‐label” foods by using sustainable marine and fungal resources to meet consumer preferences for natural, nutritious savory ingredients.

## Author Contributions

Muhammad Yusuf contributed to conceptualization, methodology, project administration, and funding. Sidney Akumu Odongo was responsible for data curation, formal analysis, writing, and software visualization. Siti Zaharah Rosli contributed through validation, investigation, resource provision, and reviewing and editing the manuscript.

## Funding

This research was funded by the FORCESS+ and the Management and Science University (Research Grant MIRG‐006‐022025).

## Ethics Statement

Sensory evaluation was conducted as part of an educational food product testing activity involving adult student participants. Participation was voluntary, and all participants were informed about the purpose and procedures of the study and provided their consent before participation. No personally identifiable or sensitive information was collected, and all data were analyzed anonymously.

## Conflicts of Interest

The authors declare no conflicts of interest.

## Data Availability

The data that support the findings of this study are available from the corresponding author upon reasonable request.
